# The Response of Human Thermal Sensation and Its Prediction to Temperature Step-Change (Cool-Neutral-Cool)

**DOI:** 10.1371/journal.pone.0104320

**Published:** 2014-08-19

**Authors:** Xiuyuan Du, Baizhan Li, Hong Liu, Dong Yang, Wei Yu, Jianke Liao, Zhichao Huang, Kechao Xia

**Affiliations:** 1 Key Laboratory of Three Gorges Reservoir Region's Eco-Environment, Ministry of Education, Chongqing University, Chongqing, China; 2 National Centre for International Research of Low-carbon and Green Buildings, Chongqing University, Chongqing, China; University of South California, United States of America

## Abstract

This paper reports on studies of the effect of temperature step-change (between a cool and a neutral environment) on human thermal sensation and skin temperature. Experiments with three temperature conditions were carried out in a climate chamber during the period in winter. Twelve subjects participated in the experiments simulating moving inside and outside of rooms or cabins with air conditioning. Skin temperatures and thermal sensation were recorded. Results showed overshoot and asymmetry of TSV due to the step-change. Skin temperature changed immediately when subjects entered a new environment. When moving into a neutral environment from cool, dynamic thermal sensation was in the thermal comfort zone and overshoot was not obvious. Air-conditioning in a transitional area should be considered to limit temperature difference to not more than 5°C to decrease the unacceptability of temperature step-change. The linear relationship between thermal sensation and skin temperature or gradient of skin temperature does not apply in a step-change environment. There is a significant linear correlation between TSV and Q_loss_ in the transient environment. Heat loss from the human skin surface can be used to predict dynamic thermal sensation instead of the heat transfer of the whole human body.

## Introduction

Thermal comfort research in buildings has primarily focused on steady-state conditions [1]–[10], while the thermal environment is often transient and dynamic over time (e.g., when moving indoor from outdoor or moving from indoor to outdoor, and taking a plane, train, boat where air-conditioning is most frequently used to adjust thermal environment to accommodate hot or cold climate). Neither ASHRAE standard 55-2010 [11] nor ISO 7730 [12] give detailed description on thermal comfort in transient environment. Increasingly, thermal comfort in transient conditions has been a focus of research [13]–[19], to further understand human response to environmental change, to improve design of building interiors and to improve thermal comfort.

Gagge et al [20] identified the important phenomena of ‘overshooting’ in thermal sensation in the step-down conditions when moving from warm to neutral and the ‘hysteresis’ in the step-up conditions when moving from neutral to warm and cold. A similar response was found from neutral to warm by the other researchers [21]–[25]. de Dear et al [21] examined the changes in overall thermal sensation in step-down and step-up conditions. The overshoot of thermal sensation was also discovered, and experimental findings were simulated by a simple thermo receptor model coupled to a numerical model of heat transfer through clothing and cutaneous tissue. Nagano et al [22] studied the thermal comfort requirements for temperature steps by experiments from hot to neutral, and confirmed that it takes more than 50 min to achieve the steady state. An immediate change in sensation after a change of environment was identified by Parsons [26] and a transient PMV model was proposed. Lomas, a researcher in German, studied temperature step change (warm-neutral) by numerical simulation [27]. Therefore, based on existing research, it may be concluded that when moving from one environment into a new one, thermal sensation in the new environment is affected by the one in previous environment. Subjects wore a clothing ensemble typical in summer in all these research.

Physiology response to temperature step has been studied by a number of researchers, and the results show that skin temperature cannot reflect thermal sensation during the transient process, although skin temperature correlated best with thermal sensation (r = 0.6) compared with skin moisture and trans-epidermal water loss (TEWL) (r = 0.42–0.54) when the temperature down-step reached 8°C [24], [28]. Ring and de Dear [29] found that magnitude estimates of cold and warm sensations caused by stimuli applied by thermodes (point sources of controlled heat) to the skin of the left thenar eminence (palm) of human subjects were proportional to the cumulative total of thermo receptor impulses during the first 20 seconds after stimulus onset. This integral was named Psycho Sensory Intensity - PSI.

From the above introduction, we can see that there are few studies on thermal response to cool-neutral-cool process when occupants wear a clothing ensemble typical in cold winter. Most researchers focused on step change in hot summer (hot-neutral and vice versa). They reported thermal response phenomenon and which physiological parameters relate to thermal sensation significantly. Only Parsons [26] proposed a modification to PMV model for evaluation on step change transient environment. His modification to PMV needs further validation. There is still a lack of studies on prediction of thermal sensation in step-change environment. We have studied the effect of step-change in temperature (warm-neutral-warm) on human thermal sensation and skin temperature and prediction of thermal sensation in hot summer, which has been reported in another paper [30]. The purpose of this paper is to determine the effect of step-change in temperature (cool-neutral-cool) in cold winter, and to explore the prediction of thermal sensation and probability of thermal acceptability. Combining study on cool-neutral-cool in this paper with the warm-neutral-warm process [30], the study on step-change will be more completed.

## Method

### Ethics Statement

The study was strictly compliant with the Declaration of Helsinki. Both the study and the consent procedure were approved by the Ethics Committee of Ministry of Health, Chongqing. Written informed consent was obtained from the participants.

### Experiments in Climate Chamber

#### Instruments

Both questionnaire surveys and human physiological measurements were used in this study. The experiments were carried out in the climate chamber in Chongqing University with human thermal sensation and thermal acceptability recorded during the temperature step process in winter of 2013. The Thermal Comfort Monitoring Station (LSI, Italy) was used to measure the environmental parameters around subjects and was located 50 cm from the subjects at a height of 60 cm, equivalent to stomach height of a sitting person. Human skin temperature was measured with copper-constantan thermocouples equipped in the multi-channel physiological acquisition system (MP150). During measurement, thermocouples were fixed to skin sites with a medical ventilator adhesive bandage. The formula of Gagge/Nishi-8W [31]was used in calculating the human mean skin temperature. Local skin temperatures were measured at eight points, including forehead, chest, back, upper arm, forearm, hand, thigh, and calf [31]. Thermal sensation was investigated with the ASHRAE 7-points scale.

#### Experimental conditions

Different cool conditions were created in the climate chamber (4 m×3 m×2.7 m, Room 1, and a neutral environment was created by air-conditioning in next door observing room (4 m×3 m×2.7 m, Room 2), both of which could be individually controlled for environmental variables.

A typical uniform clothing combination (1.17 clo) [32] was adopted for subjects to avoid the effect of difference in clothing insulation. During the experiment, the sedentary subjects could read newspapers and magazines (M = 1met) [33].

According to the Code for Design of Heating, Ventilation and Air Conditioning of Civil Buildings GB50736-2012 [34], indoor design temperature, relative humidity and air velocity in winter are recommended in the range of 18–24°C, 30%∼60%, 0–0.2 m/s. Preliminary experimental results showed that neutral temperature was 22°C when subjects wore the above uniform clothing combination in Room 2. Temperature steps of 5°C,7°C,10°C were created between Room 1 and Room 2 where temperature of the cool environment was controlled as 17°C, 15°C, 12°C in Room 1. RH was controlled at approximately 50% in the experiment. To make the indoor velocity field uniform, a perforated ceiling air supply with side air return was adopted in climate chamber, with an air velocity less than 0.1 m/s to avoid cold air draft. Experiments were carried out in winter. The experimental conditions are detailed in Table 1 and Table 2.

**Table 1:** Experiment conditions-Room 1.

Design condition	12-22-12°C	15-22-15°C	17-22-17°C
Ta	12.16±0.05	15.16±0.13	16.95±0.02
RH	57.41±0.67	58.20±0.73	54.31±0.36
V	0.071±0.007	0.032±0.004	0.062±0.005
Tg	12.10±0.04	14.83±0.04	16.58±0.04
Tr	12.95±0.06	14.71±0.06	16.51±0.15
To	12.08±0.06	15.00±0.07	16.64±0.12

Note: Values in the table in mean value ± standard deviation.

Ta, RH, V, Tg, Tr, and To in Table 1 represent respectively air temperature, relative humidity, velocity, black global temperature, mean radiant temperature, and operative temperature.

**Table 2:** Experiment conditions-Room 2.

Design condition	12-22-12°C	15-22-15°C	17-22-17°C
Ta	22.22±0.07	22.23±0.05	22.54±0.05
RH	43.92±0.30	51.01±0.32	49.10±0.22
V	0.015±0.003	0.003±0.001	0.003±0.001
Tg	21.75±0.12	21.26±0.06	21.28±0.06
Tr	22.10±0.22	21.21±0.06	21.21±0.07
To	22.00±0.06	21.47±0.05	21.91±0.04

Note: Values in the table in mean value ± standard deviation.

Ta, RH, V, Tg, Tr, and To in Table 1 represent respectively air temperature, relative humidity, velocity, black global temperature, mean radiant temperature, and operative temperature.

#### Subjects

Some researchers have studied on effects of gender on thermal perception. Dissatisfied percentage of females outweighs males in both cold and hot environment [34], [35]. Females are more sensitive on environmental temperature than males [36]–[38]. Thus thermal response of males and females on step change should be studied separately and compared. Males are the focus of this paper. Females will be studied in next step. The subjects who participated in the experiments were volunteers. To avoid effect of age and social back ground on the results, subjects were required to be healthy and aged 20 to 30 years old with bachelor degree or above. To ensure experiments were carried out smoothly and to ensure validity of experimental data, no strenuous exercise or cold, nor feeding or drinking was allowed within 24 hours and the subjects had a good sleep just before the experiment. During the experiment, the subjects were only allowed to do light reading or rest during the experiment, to keep their moods as stable as possible during the experiment. Finally, 12 male subjects participated in the experiment.

#### Experimental Procedures

All subjects were required to participate in all the three experiments. Each experiment lasted for 2 hours. The time intervals were based on preliminary experiments for the three conditions.

For each experiment condition, uniform clothing was put on and thermocouples were fixed on skin sites after each subject had arrived at Room2. Subjects were then kept sedentary until the skin temperature was stable and thermal sensation was voted as 0. This took approximately 30 minutes. Sedentary activities (reading) were allowed. Then the subject entered the cool environment Room 1 and the experiment formally began. Thermal sensation survey, thermal comfort survey and skin physiology evaluation were performed simultaneously. Subjects were asked to report their impressions of the environment in the chamber every 2 minutes for 10 minutes and then every 10 minutes for 20 minutes by responding to a questionnaire. After 30 minutes period, the subject moved into the neutral environment (Room2) and recorded thermal sensation immediately when he came out of Room 1. Next, he would undergo 60-minute test period. During the period, subjects recorded thermal sensation every 2 minutes in the early 10 minutes, then once for every 10 minutes for the rest 50 minutes. After that subjects were asked back to Room 1 for 30 minutes during which they were required to vote once for 2 minutes in the early 10 minutes and once for 10 minutes for the rest 20 min. In the whole process of experiment, the skin temperature was collected every minute.

### Experiments in Offices

In March, 2010, step change experiments in daily life were carried out in three air-conditioned offices and one office without air conditioning. Three neighboring offices were used, each with the same building envelope and area. They were located on the same floor. The air-conditioners in the three offices were the same, and they were Gree air-conditioners (KFR-50LW/K(5057L)-N5). These conditions insured that experimental conditions in the three offices were consistent as much as possible. The office without any air-conditioning was located opposite of the three air-conditioned offices.

According to the Code for Design of Heating, Ventilation and Air Conditioning of Civil Buildings GB50736-2012 [34], indoor design temperature, relative humidity and air velocity in winter are recommended in the range of 18–24°C, 30%∼60%, 0–0.2 m/s. Therefore, 18, 20, 22°C were set as the experimental temperature. The experiments were carried out in March, 2010. To simulate working in offices, the experiments were carried out between 9.00 am∼6.00 pm. The indoor environmental parameters during the experiments are shown in Table 3. These were measured by the same instruments – the Thermal Comfort Monitoring Station (LSI, Italy) introduced in Section 2.2.1.

**Table 3:** Indoor environmental parameters.

Room	Temperature/°C	Relative humidity/%	Air velocity
No air conditioner	13.3±0.56	71.4±5.07	0.062±0.007
Air conditioner, 18°C	17.7±0.19	59.8±1.19	0.068±0.003
Air conditioner, 20°C	20.2±0.19	53.2±1.10	0.076±0.003
Air conditioner, 22°C	22.4±0.22	47.3±1.73	0.066±0.002

Note: Values in the table in mean value ± standard deviation.

To avoid effect of age and social back ground on the results, subjects were required to be healthy and aged 20 to 30 years old with bachelor degree or above. To ensure experiments were carried out smoothly and to ensure validity of experimental data, no strenuous exercise, nor feeding or drinking was allowed within 24 hours just before the experiment, and the subjects were only allowed to do reading or rest during the experiment, to keep the subjects' moods as stable as possible. 20 subjects (10 males and 10 females) participated in the experiments. The requirements used for choosing the subjects are detailed in section 2.2.3. The aim of the experiments was to simulate a real step change process, so subjects wore their own clothes in the experiments. Mean cloth insulation of subjects in the experiments was 1.14clo (±0.11clo). Subjects rested for 30 minutes in the office with no air-conditioning, then entered in air-conditioned office and recorded their acceptability about temperature step change immediately. They undertook reading or writing for 30 minutes in air-conditioned office, then return into the office with no air conditioner and recorded their acceptability about temperature step change immediately. Each subject participated in experiments in all three conditions. Thermal acceptability of step change was recorded during experiments.

## Results and Analysis

The results of the subjective votes and skin temperatures were analyzed using Friedman's ANOVA test [39].

### Thermal Sensation Vote (TSV)

**Figure 1:**
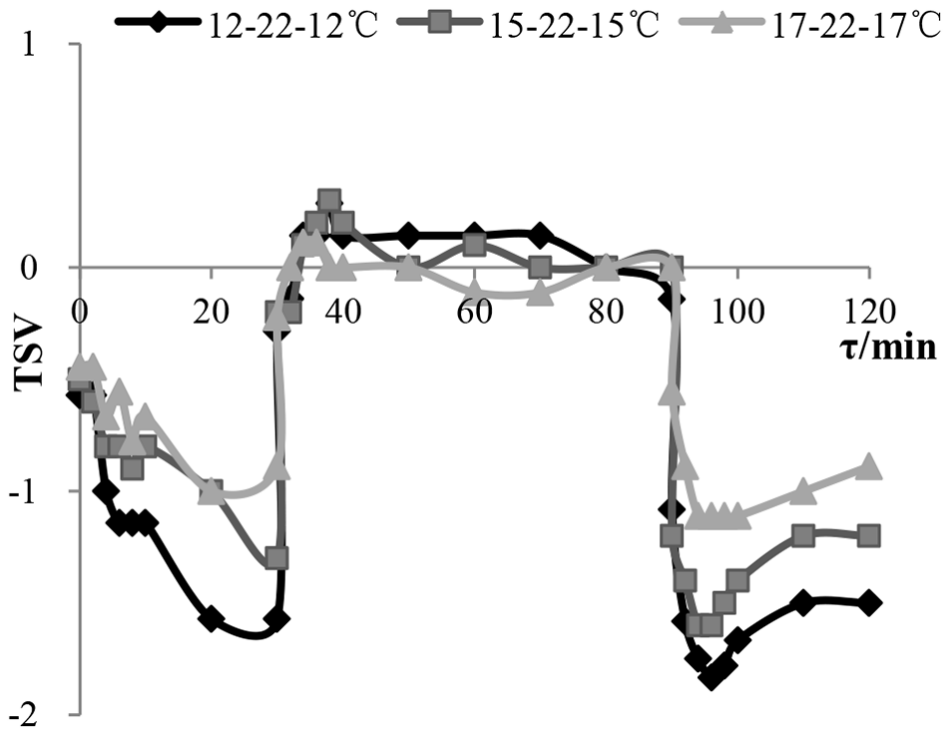
The changing process of TSV in the experiment in response to temperature step change. The vertical axis is TSV(thermal sensation vote), and the horizontal axis is time——τ (min). This figure shows TSV changing as time in the three step-change temperature conditions ——12-22-12°C (rhombus label), 15-22-15°C (square label), 17-22-17°C (triangle label).

Fig. 1 shows changes of thermal sensation vote (TSV) over time when subjects had moved between a cool environment and a neutral environment. TSV experienced a gradual decrease during the first 30 minutes, then increased sharply when subjects moving in neutral environment. Following that, a slight fluctuation of TSV occurred during the period when subjects stayed in neutral environment. Compared with the first 30 minutes, dramatic decrease of TSV appeared immediately because of step-down change. And then TSV slightly rose in the last 30 minutes. TSV was unstable in first 30 minutes and the last 30 minutes in each of the three conditions, and a significant difference of TSV occurred over time in each cool environment (p<0.05). However, there was no significant difference in neutral environment (p>0.05). It is interesting that TSV belonged to thermal comfort zone (−0.5≦TSV≦+0.5) when moving into neutral environment from cool environment. Therefore, dynamic thermal sensation model is not necessary predicting thermal sensation when moving from cool to neutral environment.

When subjects moved into neutral environment from a cool environment, overshooting of TSV occurred immediately (see Fig. 1). Due to the temperature step-up, TSV increased to an approximately neutral level in a short time and continuously rose to a peak in 4 to 8 minutes after the temperature step-up. It then reduced and kept at a neutral level. This was due to heat transfer lag because of clothing insulation. The lower the temperature in the previous cool environment, the higher the TSV in the neutral environment and the more significant the overshooting.

For subjects moving from a neutral to a cool environment, they underwent two similar transient processes (in experiment which were step-down at the 0th minute from neutral to cool environment and a step-down at the 90th back to cool environment from neutral environment). However, human thermal response to temperature step-down was unlike (see Fig. 1). TSV decreased gradually to trough in 4 minutes and then picked up slightly in the step-down at 90th minute. While no overshooting of TSV occurred in temperature step-down change at the 0th minute. The difference of the two transient processes is the thermal experience, which suggests that thermal experience has a great effect on thermal sensation.

In addition, the results of the changes of thermal sensation as time (Fig. 1) suggest that human thermal sensation presents asymmetry in the same cool thermal environment after temperature steps, in other word, thermal sensation from the neutral environment back to the initial cool environment was always lower than initial thermal sensation in the same environment.

### Mean Skin Temperature

Whether the situation is a temperature step-down or a step-up, human thermoregulation plays a role. One thermoregulation is blood-supply to the skin surface. When temperature decreases suddenly, vasoconstriction immediately induces blood transfer from skin surface and takes a mass of heat away reducing skin temperature [40]. When the temperature increases, blood transfers to the skin surface bringing heat, which increases human skin temperature.

**Figure 2:**
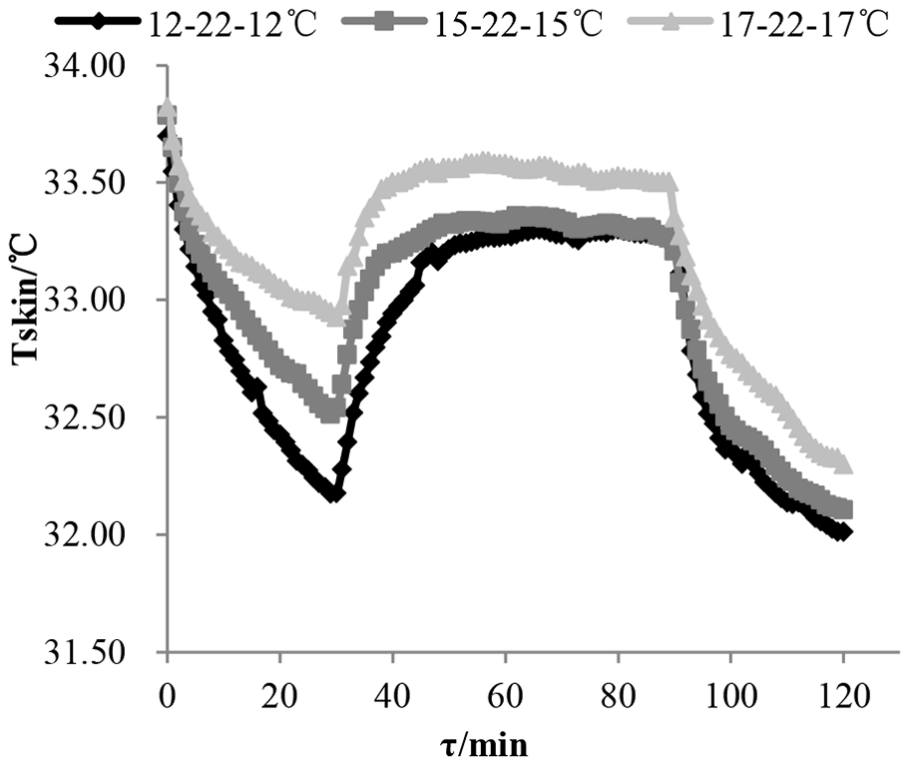
The change of human mean skin temperature over time in response to temperature step change. The vertical axis is mean skin temperature, and the horizontal axis is time——τ (min). This figure shows mean skin temperature changing as time in the three step-change temperature conditions ——12-22-12°C (rhombus label), 15-22-15°C (square label), 17-22-17°C (triangle label).

Fig. 2 illustrates the changing process of human mean skin temperature in the experiment. Overshooting did not occur in mean skin temperature. For all three cases, mean skin temperature plunges immediately in the first 30 minutes of acclimation after temperature step-down, the decreasing rate slowing gradually. Following the next temperature step-up, mean skin temperature rises sharply back to a new constant state. In the cool environment, the human mean skin temperature immediately reduces again sharply, which may be the reason for overshoot of cold sensation - overall thermal sensation was lower than when it returned in the short acclimation time after temperature step-down. It is interesting that mean skin temperature decrease due to step-down outweighed than mean skin temperature increase due to step-up.

Results tested with Friedman in first stage of the condition 22°C −12°C shows that a step change with significant difference (p = 0.006<0.05) occurred among mean skin temperatures at the 28th to 30th minutes, but no significant difference (p = 0.285>0.05) occurred between the 29th and 30th minutes. This means that mean skin temperature stabilized after 29 minutes. In a similar way, time was obtained for the mean skin temperature to stabilize (see Table 4). It can be seen that mean skin temperature needed approximately 30 minutes to stabilize gradually when subjects were moving to cool environment from neutral environment (the step change was not more than 10°C). However, Fig. 2 shows that mean skin temperature still decreased and was not stable in 30 minutes. This suggests that after 30 minutes, human mean skin temperature changed much more slowly. Whilst, for subjects from cool to neutral environment, mean skin temperature gradually became a constant after about 20 minutes when the up change was less than 10°C. As the step change reduced, the stabilizing time of mean skin temperature decreased.

**Table 4:** Time to stabilize for human mean skin temperature.

condition	stage	Period of p<0.05	Period of p>0.05	Time to stabilize
12-22-12°C	1st stage (down-step)	28–30 min (p = 0.006)	29–30 min (p = 0.285)	29 minutes
12-22-12°C	3rd stage (down-step)	112–120 min (p = 0.038)	113–120 min (p = 0.074)	23 minutes
12-22-12°C	2nd stage (up-step)	47–90 min (p = 0.046)	48–90 min (p = 0.241)	18 minutes
15-22-15°C	1st stage (down-step)	25–30 min (p = 0.017)	26–30 min (p = 0.081)	26 minutes
15-22-15°C	3rd stage (down-step)	115–120 min (p = 0.028)	116–120 min (p = 0.211)	26 minutes
15-22-15°C	2nd stage (up-step)	42–90 min (p = 0.044)	43–90 min (p = 0.095)	13 minutes
17-22-17°C	1st stage (down-step)	17–30 min (p = 0.037)	18–30 min (p = 0.052)	18 minutes
17-22-17°C	3rd stage (down-step)	114–120 min (p = 0.013)	115–120 min (p = 0.082)	25 minutes
17-22-17°C	2nd stage (up-step)	40–90 min (p = 0.028)	41–90 min (p = 0.095)	11 minutes

### The Relationship between Mean Skin Temperature and Thermal Sensation in transient environment

The change of human skin temperature is the result of skin blood flow regulation and it is an important physiological parameter reflecting heat transfer between human body and environment and a human stress response to cold or heat. Therefore, skin temperature has an effect on human thermal sensation.

According to previous studies in steady environment [20], [41], there is one to one correspondence between mean skin temperature and human thermal sensation. Mean skin temperature can predict human thermal sensation as an important parameter in a steady environment.

In step change environment (warm-neutral-warm) (Hong Liu et al, 2014), mean skin temperature is not enough to determine the comfort level of the occupants. There is a significant linear relationship between TSV and the gradient of mean skin temperature (dTskin/dτ). The gradient of mean skin temperature can be used to predict dynamic thermal sensation in step-down.

**Figure 3:**
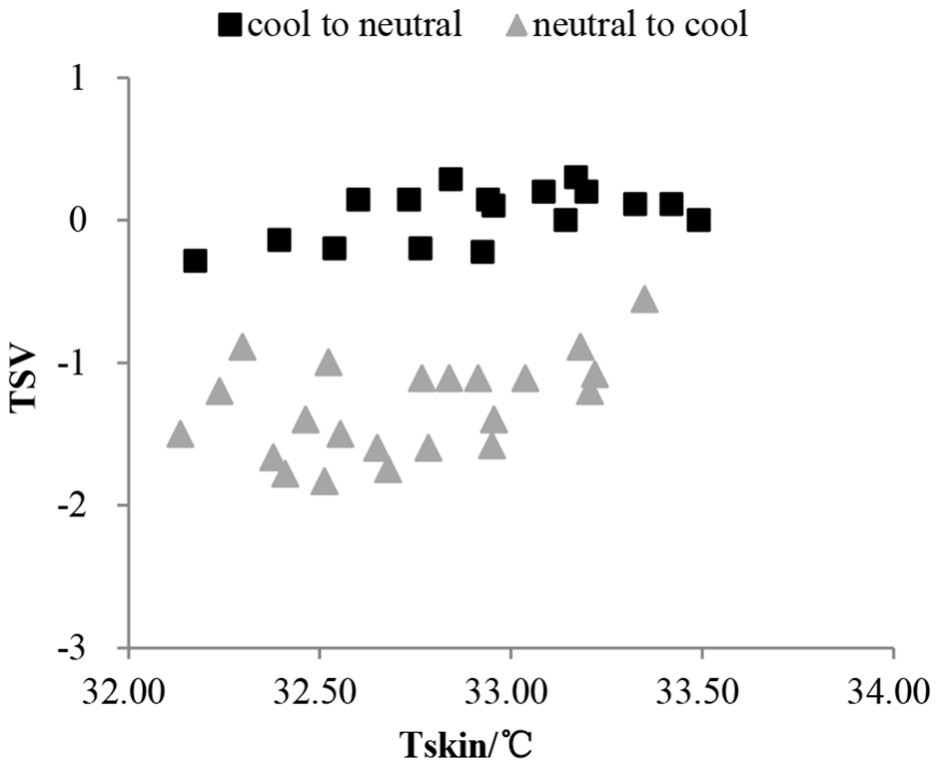
The relation between thermal sensation and skin temperature. The vertical axis is TSV(thermal sensation vote), and the horizontal axis is mean skin temperature (°C). This figure shows the relation between thermal sensation and mean skin temperature in the transient process from cool to neutral (square label) and from neutral to cool (triangle label). The transient process means step-change period till mean skin temperature stable.

**Figure 4:**
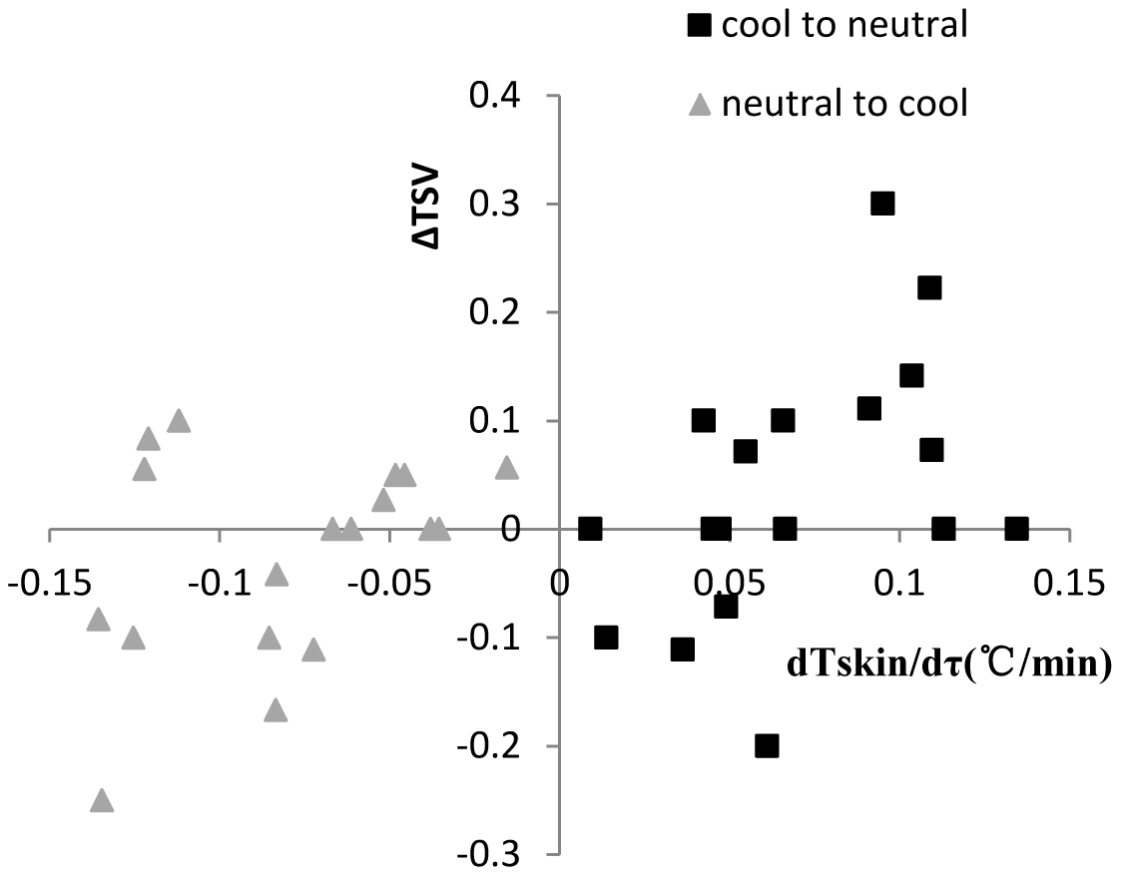
The relationship between gradient thermal sensation and gradient of skin temperature. The vertical axis is delta TSV(thermal sensation vote), and the horizontal axis is gradient of mean skin temperature (°C /min). This figure shows the relation between the gradient thermal sensation and the gradient of mean skin temperature in the transient process from cool to neutral (square label) and from neutral to cool (triangle label). The transient process means step-change period till mean skin temperature stable.

Fig. 3 shows no significant linear relationship between TSV and Tskin in step change environment (cool-neutral-cool), just like the results of Hong Liu [30]. The skin temperature and TSV shown in Fig. 3 were selected based on Table 4 as the data of transient process. Furthermore, the relation is not one to one correspondence. The same mean skin temperature was corresponded by different thermal sensations. This is different with the relationship in steady environment. In addition, no significant linear relationship existed between the gradient TSV (delta TSV) and the gradient of mean skin temperature (dTskin/dτ) (see Fig. 4). Therefore, neither the mean skin temperature nor its gradient can be used to predict the human thermal sensation in transient environment (cool-neutral-cool).

### Heat Loss from human skin surface (Q_loss_)

Heat transfer between human body and the ambient environment is very important for prediction of thermal sensation. PMV is deduced by the heat transfer of whole human body (Fanger, 1970).When people are in steady state, the heat exchanges tend towards steady. Therefore, TSV correlates significantly to mean skin temperature in linear relationship. However, it is variational and dynamic in transient environment. Based on the theory of PMV, there should be a certain relationship between TSV and heat loss from human skin surface. The computed process of Qloss has been detailed in another paper [30]. This assumption is validated in both coo-neutral-cool process and warm-neutral-warm process [30].

**Figure 5:**
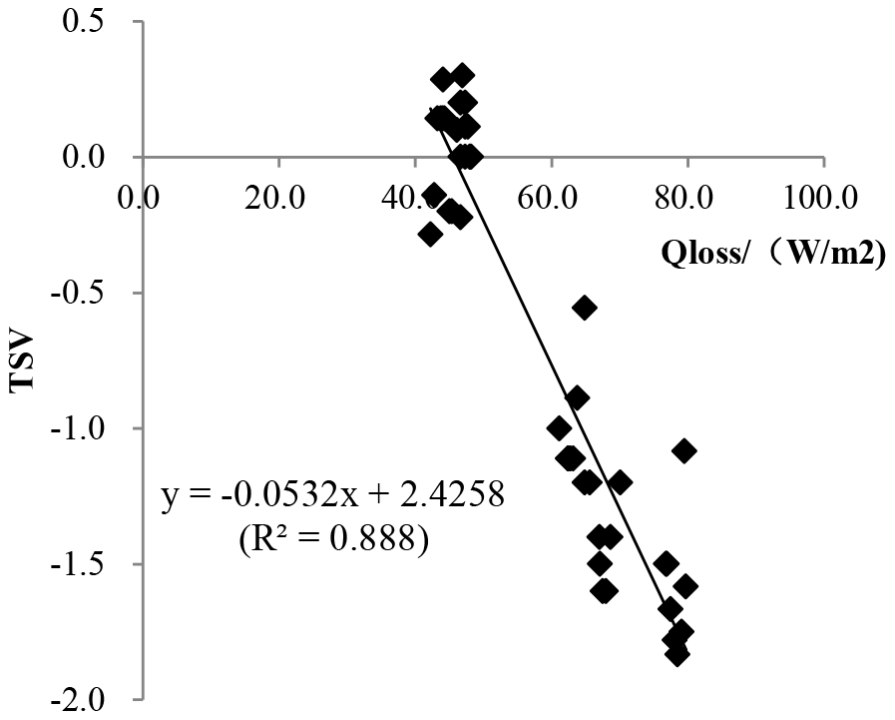
The Relationship between TSV and Heat Loss from Skin Surface. The vertical axis is TSV (thermal sensation vote), and the horizontal axis is heat loss from skin surface (W/m2). This figure shows the relation between thermal sensation and heat loss from skin surface in the transient process both from cool to neutral and from neutral to cool. The transient process means step-change period till mean skin temperature stable.

Fig. 5 shows a linear correlation was significant between TSV and Qloss in the transient environment (cool-neutral-cool) (TSV = −0.0532Qloss+2.4251, R^2^ = 0.888). The results of Hong Liu in step change experiments (warm-neutral-warm) also show a significant linear relationship between TSV and Qloss. We can conclude that Qloss can be considered as a significant index to predict human thermal sensation when people undergo temperature step change. This suggests a new idea for dynamic prediction of thermal sensation in transient environment. If researchers want to find a universal dynamic model to predict TSV fit for all temperature conditions, human heat transfer must be analyzed in theory. Compared with heat transfer between the human body and the ambient environment, heat loss from the human skin surface is much easier to be calculated. So it can be used to predict dynamic thermal sensation instead of the heat transfer of the whole human body.

### Evaluation on Acceptability of Temperature Step Change

In the questionnaire, it is reported whether the temperature step change is acceptable or not. “Acceptable” is valued as 1 and “unacceptable” is valued as 0. To study relationship between the difference in temperature of step change (temperature after step change minus the temperature in the previous environment) and acceptability, logistic regression of SPSS 20.0 is used to analyze the relation and regression equation as follows. Votes of acceptability include both data from the experiments in this paper and from experiments in 2010 [42]. The experiments in 2010 are described in ‘2.3 Experiments in offices’.
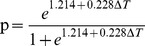
(1)Where, ΔT is temperature after step change minus the one in previous environment (°C) and p is acceptable probability.

The results of testing the logistic regression equation are shown in Table 5 and Table 6. These suggest that the difference of temperature in step change has significant effect on acceptability of step change and that Equation (1) can be applied.

**Table 5:** Verification of Hosmer-Lemeshow.

?^2^	df	Sig.
7.580	8.000	0.476

**Table 6:** Variables in the equation.

	B	S.E,	Walds	df	Sig.
ΔT	0.228	0.045	25.758	1	0.000
Constant	1.214	0.286	17.977	1	0.000

**Figure 6:**
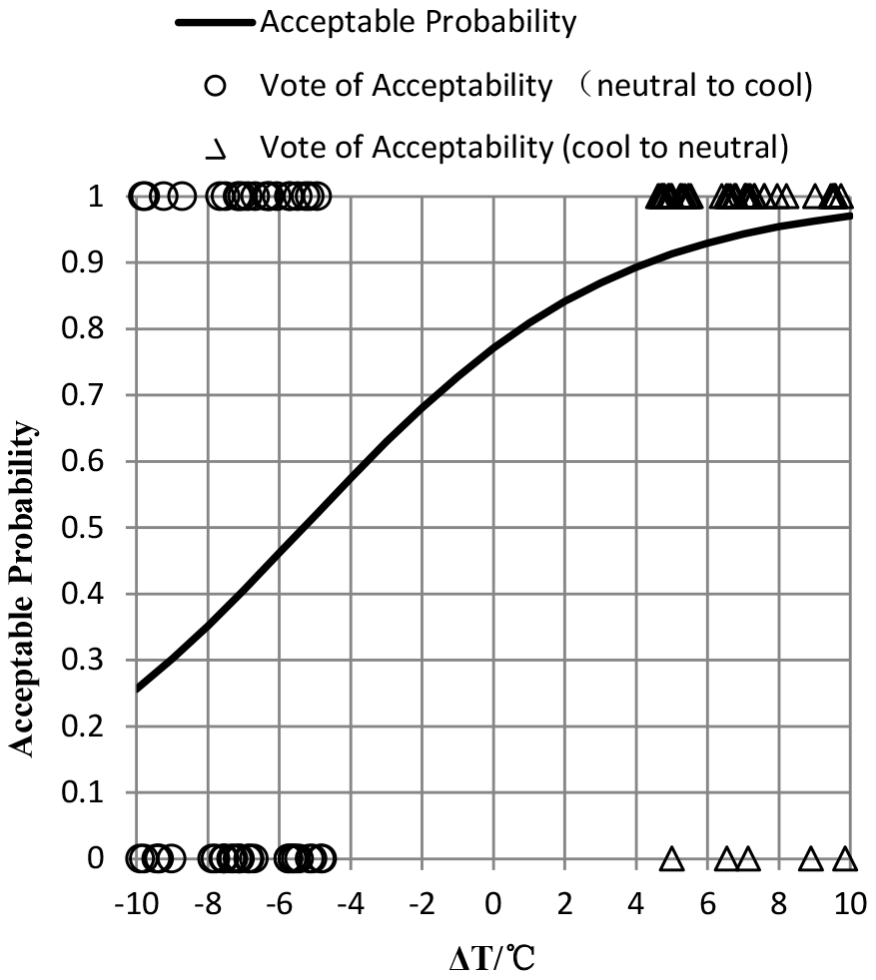
Two-dimensional logistic regression model. This figure shows the model predicting acceptable probability of temperature step change. The vertical axis is acceptable probability of the temperature step change, and the horizontal axis is ΔT (temperature after step change minus the one in previous environment). In the questionnaire, it is reported whether the temperature step change is acceptable or not. “Acceptable” is valued as 1 and “unacceptable” is valued as 0.

Fig. 6 was formed by Equation (1). When p equals 0.5, the probability of acceptability was the same as the one of unacceptability and temperature difference was 5.3°C. The probability was more than 50% in the condition of ΔT<5.3°C moving into a cool environment from neutral. When moving into a neutral from a cool environment, subjects accepted the temperature difference of step change in the range of 10°C, and the probability was greater than 80% approximately.

PMV-PPD has been applied extensively in evaluation of steady environment. It recommends the environmental parameters which ensure 80% occupants feel acceptable in the environment. This has been validated by most researchers. However, in step-change environment, no model exists to evaluate occupants' acceptable probability. This evaluation model for step change (Equation 1) in this paper should therefore be further investigated and validated. In addition, all these results in this paper therefore need further study and validation whether they can be applied on female subjects.

## Discussion

In this study, the temperature step-change concluding down-step and up-step was studied between two different ambient environments. As with other similar studies, there was an observed overshoot in thermal sensation in response to the down-step conditions, as shown in Table 7. It can be seen that overshooting of thermal sensation is widespread in experiment of temperature step change. However, the overshoot in this study is much smaller than those reported by others under different conditions. This may be attributed to different clothing insulation.

**Table 7:** Comparison among different countries' results on temperature step change.

Country	Environment	Method,	Temperature/°C	TSV	Results
Japan	Uniform	Experiment	34-28,25,22; 37-31,28,25	Hot-warm, neutral, cool	Descriptive
German	Uniform	Experiment and Numerical simulation	28-18, 27-22, 21-17	Warm-cool, neutral; Neutral-cool	Model
Australia, Denmark	Uniform	Experiment, numerical simulation	17.7–25.5, 25.4-17.6, 17.7-12, 12.8–17.6	Neutral-hot. cool; Hot. cool-neutral	Model
America	Uniform to non-uniform	Experiment	28-14	Warm-cool	Model
China	Uniform	Experiment	35,32,30, 28-25	Hot, warm-neutral	Descriptive
This paper	Uniform	Experiment	12,15,17–22	Cool-neutral	Descriptive

Researchers in Australia and Denmark [21], [29], China [30], [43], Germany [27], America [19], [25], [28] and Japan [16], [18], [22] have studied thermal comfort in step-change environment. Step-change temperatures in these previous experiments are different, and focus on the step-change passing from comfort temperature and uncomfortable temperature, or the opposite process. Step-change in uniform and steady environment is the focus of researchers in Australia, Denmark, China, Japan and Germany, whilst in America researchers have studied the local step-change caused by local stimulation which makes the environment non-uniform. Experiments have been carried out in China, Japan and America, whilst numerical simulation has been used by Australian and German researchers and is verified by experiments. Thermal sensation studied by researchers is in different ranges. Researchers in China and Japan focus on the step-change passing from warm (cool) to neutral, or the opposite process. Researchers in America, Australia and Germany focus on the step-change passing from slightly cool (slightly warm) to neutral, or the opposite process. Most of the experiments are carried out with great temperature step-change (more than 5°C). There are few discussions on small temperature step-changes. Compared with the qualitative description of the experimental results by Chinese and Japanese researchers, thermal response model in step-change environment is proposed by American, Australian and German researchers based on their own experimental results. In this paper, our research which included both up and down step processes is studied, rather than a single up or down step. Therefore, the results of the changes of thermal sensation with time (Fig. 1) suggest that human thermal sensation presents asymmetry in the same cool thermal environment after temperature steps, in other words, the thermal sensation from the neutral environment back to the initial cool environment was always lower than the initial thermal sensation in the same environment.

The experimental results differ between researchers. Mean skin temperature changes gradually and slowly when passing from warm to neutral, as found by other Chinese researchers [30], [43], whilst Japanese experiments [16], [18], [22] show that a small sharp decrease appears in mean skin temperature when passing from warm to neutral. This is due to the different endurance of the two races. Chinese endurance on temperature step-change is greater than Japanese. Hysteresis and anticipatory phenomenon as reported by Gagge in 1969 [20] appear in temperature step-change studied by the researchers. Subjects' thermal acceptability depends on their whole thermal state before the step-change and the level of temperature step range. Subjects feel unacceptable and their thermal sensation outweighs 2 (warm) when the temperature step range outweighs 5°C passing from comfort environment to uncomfortable environment. The bigger the temperature step range, the more uncomfortable. Subjects feel. When passing from an uncomfortable environment to a comfort environment, subjects feel acceptable and their minimum thermal sensation reaches −1 (slightly cool). Neutral thermal sensation in a step-change environment cannot achieve the thermal state with maximum acceptability. Perhaps 5°C is the critical temperature step range, and there is lack of thermal comfort study on step range less than 5°C. In this paper, this is verified by logistic regression. This indicates that the PMV-PPD of Fanger is not applicable to evaluate a step-change environment. However, the present evaluation standards on the thermal environment such as ASHERE Standard 55–2004 and Standard ISO-7730 are mainly applied in steady environment. Because of particularity of thermal response to step-change, the evaluation is more difficult and complex than PMV for steady environment. Evaluation models are different between up-step and down-step. In terms of transient spaces, proper temperature difference between inoperative zone and operative zone is good for human health and energy consumption far from preventing human thermal comfort.

‘Alliesthesia’ could explain psychological processing of thermal responses to a dynamic environment. Cabanac proposed the term ‘alliesthesia’ coming from esthesia (meaning sensation) and allios (meaning changed) in a landmark paper [44]. Alliesthesia depends upon internal signals. A given stimulus can induce either a pleasant or an unpleasant experience depending on the subject's internal state. Alliesthesia mediates behavioral responses (negative feedback) to their goals, which can explain behavioral adaption in the body's regulatory systems such as hunger, thirst or temperature regulation. Take temperature regulation as an example. The internal signal in temperature regulation is the difference between the real core temperature and a set value. This set value depends upon the species and is about 37°C in man. Fever is a state where the set value of the regulated body temperature is higher than it is in the healthy state. For a subject who has a fever, a warm stimulus is normally unpleasant since his temperature 37.5°C is higher than 37°C (the set point). However, if his set temperature is 38°C, a 37.5°C core temperature is below his set value and he will describe a warm skin stimulus as pleasant. Alliesthesia leads subjects to seek pleasant stimuli and avoid unpleasant ones.

**Figure 7:**
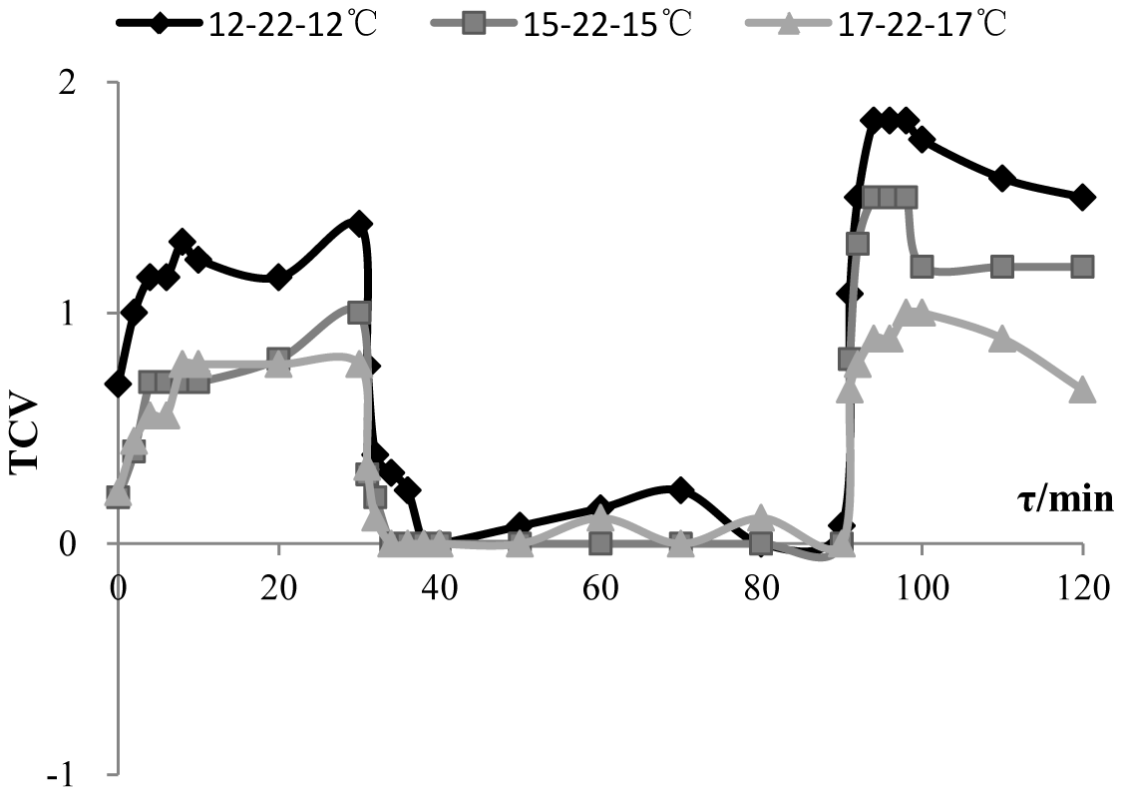
The changing process of TCV in the experiment in response to temperature step change. The vertical axis is TCV(thermal comfort vote), and the horizontal axis is time——τ(min). This figure shows TCV changing as time in the three step-change temperature conditions ——12-22-12°C (rhombus label), 15-22-15°C (square label), 17-22-17°C (triangle label). ‘0’ means comfort. ‘1’ means- a little uncomfortable. ‘2’ means uncomfortable. ‘3’ means very uncomfortable.

This paper is focused on thermal comfort rather than thermal extremes pf hyperthermia or hypothermia. Thermal sensation describes the magnitude and sign (warm versus cool) of the experience, whereas thermal comfort qualitatively describes the hedonic tone or pleasantness of the stimulus (like versus dislike) [45]. Fig. 7 shows changing process of TCV (thermal comfort vote). It can be seen from Fig. 1 and Fig. 7 that there are differences between thermal sensation and thermal comfort. No overshoot occurred in TCV during step-up (cool to neutral), whereas it occurred in TSV. Overshoot occurred in both TCV and TSV during step-down (neutral to cool). This is accordant with alliesthesia, which could explain why most subjects feel unacceptable during step-down (neutral to cool), while most subjects felt acceptable during up-step (cool to neutral). When subjects were in a neutral environment, they felt thermal comfort and a neutral status. However, when they entered the cool environment, the original comfort psychological balance was broken, and cold stimulus was unpleasant (negative alliesthesia). Therefore, most subjects felt unacceptable to this step-down. Since psychological and physical adjusting systems worked, the error between the regulated variables (core temperature and skin temperature) within the milieu interieur and their set-points decreased. Finally, a new physical and psychological regulation balance was achieved, which led to stable TCV and TSV. When subjects were originally in a cool environment, they felt uncomfortable. In this circumstance, the warm stimulus was pleasant (positive alliesthesia). Therefore, subjects felt thermal comfort and a neutral status when they entered into a neutral environment from the original cool environment.

## Conclusions

Temperature step change results in an overshoot of TSV. Thermal experience has a great effect on thermal sensation Asymmetry appears when subjects returned to the previous cool environment compared with when first entering the cool environment. When moving into neutral environment from cool environment, dynamic thermal sensation is in the thermal comfort zone (−0.5≦TSV≦+0.5) and the overshoot of TSV is not obvious.

When the temperature difference between two environments in winter is greater than 5°C, persons will feel uncomfortable and unacceptable in terms of a step-change when moving into a cooler environment from a comfortable environment with air-conditioning. Air-conditioning in a transition area is better to be considered to control temperature difference by not more than 5°C when thermal comfort with high quality takes place in special occasions (e.g. taking aircraft, or by train).

Deviation between mean skin temperature and TSV exists in a temperature step-change situation. The relationship between thermal sensation TSV and human mean skin temperature or between gradient TSV and gradient of mean skin temperature) in a transient environment is not linear as in steady environment. Heat loss from the human skin surface can be used to predict dynamic thermal sensation instead of the heat transfer of the whole human body.
